# Viral infections in fire ants lead to reduced foraging activity and dietary changes

**DOI:** 10.1038/s41598-018-31969-3

**Published:** 2018-09-10

**Authors:** Hung-Wei Hsu, Ming-Chung Chiu, DeWayne Shoemaker, Chin-Cheng Scotty Yang

**Affiliations:** 1 Laboratory of Insect Ecology, Graduate School of Agriculture, Kyoto University, Kitashirakawa Oiwakecho, Kyoto, 606-8502 Japan; 2 Department of Entomology, National Taiwan University, Taipei, 106 Taiwan; 3 Department of Biological Resources, National Chiayi University, Chiayi, 600 Taiwan; 4 Department of Entomology & Plant Pathology, University of Tennessee, Knoxville, Tennessee 37996 USA; 5 Research Institute for Sustainable Humanosphere, Kyoto University, Gokasho, Uji, Kyoto, 611-0011 Japan

## Abstract

Despite the presence of conserved innate immune function, many insects have evolved a variety of mechanical, chemical, and behavioral defensive responses to pathogens. Illness-induced anorexia and dietary changes are two behavioral defensive strategies found in some solitary insects, but little is known regarding the role of such behaviors in social insects, especially in ants. In the present study we examined if such reduced foraging activity exists for a social insect, the invasive fire ant *Solenopsis invicta*, and its viral pathogen, *Solenopsis invicta* virus 1 (SINV-1). Virus-free fire ant colonies were split into two colony fragments, one of which subsequently was inoculated with SINV-1. Four food resources with different macronutrient ratios were presented to both colony fragments. SINV-1-inoculated colony fragments consistently displayed reduced foraging performance (e.g., foraging intensity and recruitment efficiency), a decline in lipid intake, and a shift in dietary preference to carbohydrate-rich foods compared with virus-free fragments. These findings provide the first evidence for virus-induced behavioral responses and dietary shifts in shaping the host-pathogen interactions in fire ants. The findings also suggest a possible mechanism for how fire ant colonies respond to viral epidemics. Potential implications of these behavioral differences for current management strategies are discussed.

## Introduction

Social insects have evolved sophisticated defensive systems presumably as adaptive responses to pathogens commonly encountered because of frequent social contact. Both allo-grooming and secretion of antibiotic compounds are two often cited examples reported in social insects^1–4^. Conversely, some pathogens have been reported to alter host behavior to favor their own transmission and enhance their replication and virulence^5^. Studies of non-social insects have reported reduced feeding (anorexia) and changes in dietary macronutrient preference in pathogen-challenged individuals, both of which are associated with an enhanced ability to cope with pathogen infections likely via starvation of resident pathogens from essential macronutrients or trade-offs in energy allocation^6^. Whether similar defensive mechanisms involving illness-induced behavioral changes also occur in social insects, especially ants, remain ambiguous^7,8^.

In this study, we tested whether reduced foraging activity or changes in macronutrient preference occur in the highly social fire ant *Solenopsis invicta* in response to a natural viral pathogen, *Solenopsis invicta* virus 1 (SINV-1). This virus is a positive-sense, single-stranded RNA (ssRNA) virus that apparently only infects ant species in the genus *Solenopsis*^9^. This virus is believed to persist largely as a chronic and asymptomatic infection but may become virulent and result in illness and colony death when ant colonies are stressed^10^. More specifically, recent studies demonstrate this virus leads to high larval mortality under certain laboratory rearing conditions and significant weight loss of queens during colony founding^11^. Several immune-related genes are up-regulated in SINV-1-infected fire ants^11^, further suggesting the virus imposes significant fitness costs under certain stressful conditions.

The discovery of SINV-1 in invasive populations of *S*. *invicta* (e.g., Taiwan^12^) offers an excellent opportunity to investigate the role of pathogen-induced behaviors in colony survival of *S*. *invicta*, especially during the initial colony founding stage when queens are known to experience significant stress as a result of starvation and exposure to harsh environmental conditions. *S*. *invicta* are opportunistic omnivores that feed on a wide variety of food resources including arthropod preys, hemipteran honeydew and plant materials, etc^13,14^. Despite great variations in their dietary preference, *S*. *invicta* have been generally regarded as oil-loving ants^14,15^, and, based on such behavior, oil has been impregnated in conventional bait products a phagostimulant to stimulate stronger feeding/foraging activity of fire ants and thus to achieve proper control^15^. Since knowledge on interplay among feeding stimulant, foraging patterns and dietary preference plays a critical role in success of bait-based management in fire ants^16^, demonstration that SINV-1 induces reduced foraging activity or changes in macronutrient intake are of critical importance because alterations of feeding rates and dietary preferences may impact the efficacies of poison baits used for fire ant control and population monitoring methods.

## Results

Results from our behavioral assays revealed significant differences in foraging patterns and macronutrient preferences between the uninfected and SINV-1-infected colony fragments. While the absence of a queen was associated with reduced foraging performance in this study, the impacts of viral infection outweighed the effects associated with queen presence and played a critical role in regulating foraging patterns of *S*. *invicta*, especially foraging intensity and recruitment efficiency.

### Foraging patterns

**Figure 1:**
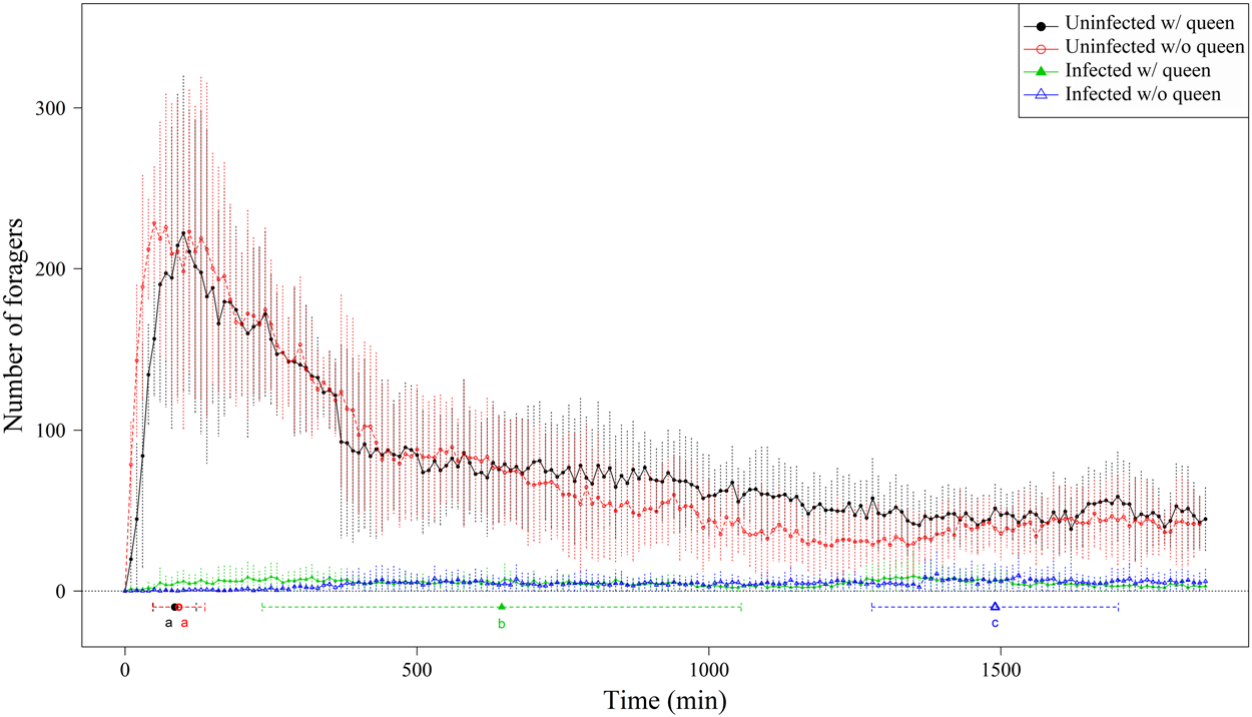
Foraging activities and peak recruitment time of uninfected (round) and SINV-1-infected (triangle) colony fragments. The open and closed symbols denote the presence and absence of a queen in a fragment, respectively. The letters (a–c) indicate significant pairwise differences (multiple comparisons conducted using pairwise *T* test, *P* < 0.05) across the four fragment categories.

Numbers of workers foraging on food resources were higher in uninfected fragments compared with SINV-1-infected fragments. Both SINV-1 infection and the absence of a queen significantly reduced the total number of forager workers, but the former had a greater impact (Fig. 1; Table 1). A similar pattern was observed for recruitment efficiency. Uninfected fragments reached peak foraging activities significantly faster than SINV-1-infected fragments. The time required to reach foraging peak significantly differed among the four fragment categories (Fig. 1, Appendix S1: Table S3, ANOVA: *F* = 21.96, df = 3, *P* < 0.001). The time required for foraging onset was not significantly different among fragment categories (Appendix S1: Table S3, Kruskal–Wallis test: *X*^2^ = 5.00, df = 3, *P* = 0.1716).

**Table 1:** Fixed effects of “virus infection” and “presence of the queen” on foraging behaviors.

Source	d.f.	*X* ^2^	*P*
**Foraging intensity**			
Queen	2	52.007	<0.001***
Infection	2	3507.8	<0.001***
Queen × Infection	1	0.131	0.7174
**Food preference**			
Food type	3	120.99	<0.001***
Queen × Food type	4	340.23	<0.001***
Infection × Food type	4	7022.2	<0.001***

Workers began to forage as soon as 17.5 ± 9.57 mins after being offered food resources in uninfected colony fragments with a queen. The recruitment efficiency in these fragments peaked at 85 ± 39.79 mins. Approximately 400 mins after food offered, the numbers of the workers decreased and stabilized at ca. 50% of the peak number (Fig. 1, Appendix S1: Table S3). Uninfected colony fragments lacking a queen displayed similar patterns in both onset of foraging (18.00 ± 13.04 mins) and recruitment efficiency (92.00 ± 44.94 mins). Queen presence had no significant effect on recruitment efficiency in uninfected fragments (Fig. 1, Appendix S1: Table S3).

Workers from SINV-1-infected fragments showed significant differences in foraging intensity compared with uninfected workers. The number of foraging workers from the former was significantly lower than that from uninfected fragments (presence or absence of a queen had no effect) (Appendix S1: Table S3). The onset of foraging in SINV-1-infected fragments also was delayed (310 ± 504.58 mins) but not statistically significant from the other three fragment categories. SINV-1-infected fragments reached a peak in foraging significantly later than uninfected fragments (622 ± 501.47 mins) (Fig. 1). We detected no effect of queen presence on the number of workers or the time of the foraging onset (135.00 ± 113.58 mins with queen; 1452 ± 169.39 mins without queen) in SINV-1-infected fragments.

### Food preferences

**Figure 2:**
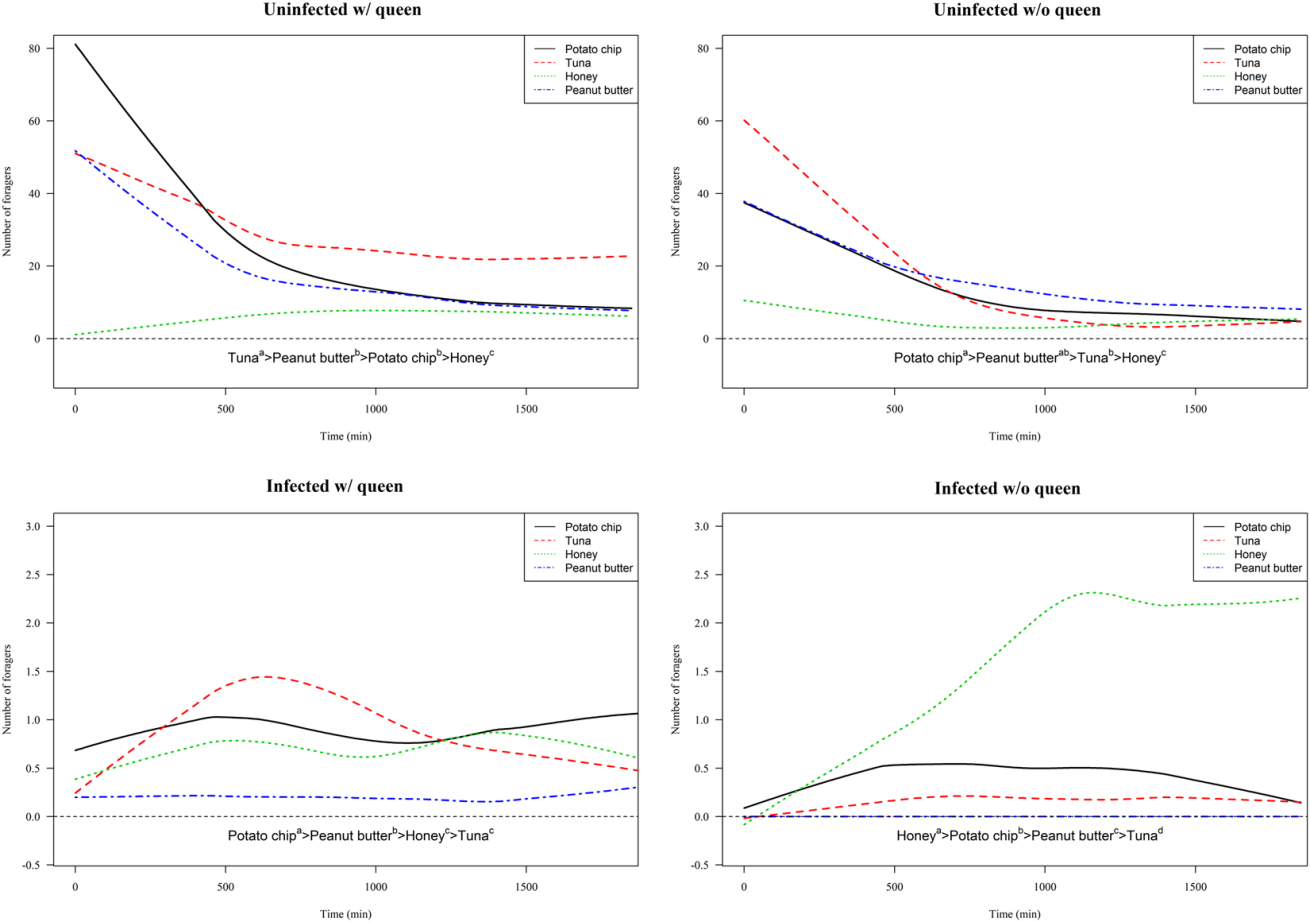
Food preference expressed as the number of workers at each food resource across time. Letters (a-d) indicate significant pairwise differences among each of the four food types across the four fragment categories (multiple comparisons conducted using a Tukey’s honestly significant difference test under generalized linear model, *P* < 0.05). Note quantitative scales for the y-axis differ among the graphs due to differences in number of foragers between uninfected and infected fragments.

Analyses of numbers of workers on different food resources indicated workers from uninfected fragments prefer high lipid or protein content food (i.e., tuna and peanut butter). In contrast, workers from SINV-1-infected fragments significantly preferred diluted honey or potato chip compared with those from uninfected fragments (Fig. 2; Table 1). Moreover, the presence of a queen influenced colony food preferences. All uninfected queenright fragments significantly preferred tuna, a food resource with relatively higher lipid and protein content (Fig. 2; Table 1).

## Discussion

Our results provide evidence for both reduced foraging activity and a change in macronutrient preferences of virus-challenged fire ants. SINV-1-infected ants invariably displayed reduced foraging activities compared with uninfected ants. SINV-1-infected ants also altered their feeding preferences toward a carbohydrate-rich diet. Similar findings were reported in fire ants infected with another single strand-RNA virus (SINV-3)^17,18^, suggesting such behavioral alterations might be common in the ant host challenged with a viral infection. Potential adaptive advantages and the illness consequences of these observed virus-induced changes in foraging behavior are discussed below, followed by the potential management implications of our results.

SINV-1 replicates in the gut epithelium of *S*. *invicta* and spreads among nestmates largely through trophallaxis^10^. Reductions in foraging activity of infected workers may lead to decreases in intra-colony food transfer and exchange, which in turn could reduce SINV-1 transmission among nestmates. Reduced social interactions as a result of decreased foraging activity have been documented in honey bees^1,19^. For example, injection of lipopolysaccharides into honey bees resulted in appetite loss and a reduction in social interactions, and both of these behavioral changes may contribute to reducing potential pathogen transmission. While such behavioral changes are due to direct virus-induced fitness costs or tradeoffs between immune response and energy allocation remains unclear, reduced social interactions and illness-induced anorexia in diseased colonies could be advantageous (e.g., less trophallaxis and brood tending) by reducing potential for spread of infections^3,20^. We suggest that the fire ant-virus system is an ideal candidate for future empirical testing of how ant hosts cope with viral infection through illness-mediated behavioral responses, which has never been tested before simply because most previous studies have focused on fungal and/or bacterial infections^21^.

Reduced foraging activities of workers from SINV-1-infected *S*. *invicta* colonies also may reduce direct exposure to interspecific competition with co-existing ants, potentially increasing their survival likelihood as a result of avoidance of interference interactions with native ants. SINV-1-infected *S*. *invicta* colonies are significantly less competitive than their uninfected counterparts when exposed to interspecific competition with native ants (i.e., lower aggressiveness)^22^. A recent field study found fire ant workers significantly outnumber workers of other native ant at the baits placed at sites with low SINV-1 prevalence, whereas fire ants account for an extremely low proportion of ants at the baits at sites with high SINV-1 prevalence (Hsu and Yang unpublished data). These field survey results, coupled with our findings, are consistent with predictions of the “resource dominance-parasitoid vulnerability trade-off theory” previously proposed by Feener^23^. In this case, local coexistence of ant species within communities likely is influenced by highly specialized pathogens, such as SINV-1, that alter interspecific interactions between competing ants via changes in behaviors of dominant species such as *S*. *invicta*. From an applied point of view, these results further suggest the biocontrol potential of SINV-1, and increasing field prevalence of SINV-1 (e.g., artificial inoculation^18^) may practically suppress fire ants both through alterations of the foraging activities and disruption of the competitive dominance of fire ants over native ants.

Reduced foraging activity of SINV-1-infected *S*. *invicta* could be among one of several illness consequences associated with this viral infection. Recently, Benaets *et al*. found that the deformed wing virus (DWV), a ssRNA virus, seemed to be responsible for reduced foraging behaviors of honey bees^24^. Moreover, most insect-infecting ssRNA viruses (including SINV-1) replicate in the intestinal epithelium^10^, raising the possibility that increased numbers of viral particles impair digestive capabilities of the epithelium, resulting in loss of appetite and reduced foraging activity^19^. Our preliminary data are partially in line with this prediction as the number of apoptotic cells in the mid-gut of the 4^th^ instar larvae of *S*. *invicta* increases with time after challenge with SINV-1 (Hsu and Yang, unpublished data). Because larval conditions in a colony can act as major drivers of worker foraging activity^25^, the reduced foraging performance of workers in our study may be indicative of changes in larval health or status (e.g., larval mortality^10^), or decreased hunger levels associated with widespread SINV-1-induced apoptosis in the intestinal epithelium.

Previous studies have reported illness-induced alterations in macronutrient intake, observed as an increase in carbohydrate intake, as a process of self-medication in multiple invertebrates after being challenged with a parasite or pathogen^6,26^. Despite the importance of innate immunity in prevention of subsequent infection in insects, increasing numbers of studies suggest that up-regulation of anti-viral immune systems is linked to a higher mortality due to energy costs^6^. Thus, compensatory feeding may be a less costly strategy as the fitness cost can be alleviated through either enhanced tolerance of microbial infections or recouping of the resources loss as a result of combating infections^27,28^. Although our data do not allow us to distinguish whether altered feeding preferences towards a carbohydrate-rich diet result from self-medication or compensatory feeding, the latter may be a more plausible case scenario. Previous studies suggest that a therapeutic behavior must meet four essential criteria, one of which predicts a decrease in fitness of an uninfected individual if engaging in a therapeutic behavior^28^. Carbohydrate supplementation is consistently associated with enhanced fitness at both the individual and colony levels in numerous ant species including fire ants^29,30^. Also, a recent study reported multiple benefits of a high-carbohydrate diet to immunity function in the ant *Ectatomma ruidum*^31^. Thus, increase of carbohydrate intake in pathogen-challenged ants may be much more prevalent than previously thought and may play a role in carbohydrate resource exploitation of ecologically dominant invasive ants.

Our results have implications for current pest management strategies of fire ants and other invasive ants. A prevailing control method of fire ants is the use of poison baits. Our data raise the interesting possibility that conventional baits may be less effective against SINV-1-infected ants as a result of changes in colony feeding patterns. First, the declining foraging activities of infected ants could lead to recruitment of fewer ants to toxic baits. Second, conventional baits may be less attractive to SINV-1-infected fire ants because of a shift in diet preferences towards carbohydrate-rich food sources. This is because current baits are impregnated with soybean oil, which serves as a phagostimulant. One predicted result of the dietary changes towards carbohydrate-rich food sources and away from food sources containing high lipid content (e.g., tuna and peanut butter) is reduced bait efficacy. In support of this prediction, a recent laboratory trial showed that SINV-1-infected *S*. *invicta* are significantly less susceptible to two chemicals commonly used in baits (0.73% hydramethylnon and 0.0103% fipronil) compared with non-infected ants^32^. While the underlying mechanism is unknown as no further tests were conducted, the behavioral and dietary changes associated with SINV-1 infections represent a potential avenue for future study. Alterations in food preferences also may help explain the generally higher prevalence of SINV-1 in many introduced areas where conventional baits have been applied broadly^12^, given these baits likely are more effective in eliminating uninfected colonies in the field. While our results are suggestive yet consistent with our prediction, additional studies are warranted to determine whether pathogen-induced phenotypic changes do indeed influence the efficacy of fire ant monitoring and management programs.

## Methods

### Colony identification, fragment formation and virus inoculations

Fire ant colonies were collected from northern Taiwan. The social form and infection status of colonies with respect to three *S*. *invicta* viruses (namely SINV-1, SINV-2 and SINV-3) were determined using diagnostic PCR assays described in detail elsewhere^33,34^. Colonies were maintained at standard rearing conditions of 28 ± 1 °C, 70 ± 10% RH and a photoperiod of 12:12 (L:D). Virus-free monogyne colonies (n = 9) were separated into two colony fragments, each consisting of 3 g of workers (nearly 3,000 individuals) and 0.02 g of brood. Note that polygyne colonies were not included in this study due to scarcity of virus-free colonies of this given social form in the field^12,35^. Fragments were maintained on a diet of frozen crickets, sucrose solution and water. The single mother queen was randomly assigned to one of the two fragments (Appendix S1: Table S1) as the presence of queen may play a role in shaping colony foraging motivation^36^. Equivalent viral titers (10^9^ genome equivalents/ml) of SINV-1 were artificially inoculated into one of each pair of colony fragments. Inoculations were done via feeding viral suspension mixed with 10% aqueous solution of honey for 24 hours^37^. Behavioral assays were conducted 30 days post inoculation. SINV-1 infection status of each fragment was monitored using RT-PCR every week after the inoculation until the end of the experiments.

### Behavioral assays: foraging patterns and changes in food preference

Different food resources with varying macronutrients, which are commonly used for monitoring of ant diversity in the field^38,39^, were provided to each colony fragment to examine the effects of SINV-1 infections on ant foraging patterns and macronutrient preferences (Appendix S1: Table S2). Each food resource was placed in a single container coated with fluon and connected independently to the colony fragment using a plastic tube (1 m). To quantify the foraging intensity, we recorded total numbers of foragers at all four food resources using an automatic recording system (camera Olympus TG-3, Olympus Corp., Japan) that takes a picture every 10 minutes for 32 hours. These pictures were analyzed to estimate the total number of foraging workers at each time point using a naked-eye-counting strategy. Time required for the first five workers to arrive at one of the four food resources was measured (onset of foraging). We also measured recruitment efficiency, defined as the time elapsed until peak in foraging was reached during a 32-hour observation period. Food preference was determined by assessing the number of workers at each food resource in both uninfected and infected fragments.

### Statistical analyses

All the colony fragments (a total of 18 fragments from 9 colonies) were divided into one of four categories based on infection status/presence of queen: uninfected w/queen (n = 4), uninfected w/o queen (n = 5), infected w/queen (n = 5), and infected w/o queen (n = 4). The effects of viral infection on fire ant foraging behavior were analyzed by comparing foraging patterns and food preferences between uninfected and SINV1-infected fragments.

For the statistical analysis of foraging intensity, defined as total number of forager workers observed on all four food resources, the effects of infection and the presence of queen were modeled by using generalized linear mixed models (GLMMs) with Poisson errors and negative-binomial errors. Despite nearly identical results, we only showed the results estimated with negative-binomial errors because of lower Akaike information criterion (AIC) values and overdispersion (Chi-square test). The numbers of ants at each time point are considered as response variables, infection status and presence of queen as fixed effects, and colony identity and each time point as random factors. The full model was built by “lme4” package in *R* (*R* Development Core Team, 2014) with the function, “glmer”. Significances of the fixed effects were tested using the likelihood ratio test by comparing the full model and models sequentially removing each effect term. The post hoc multiple comparisons of the four fragment categories was conducted with Tukey’s all-pairwise comparisons implemented in the *R* package “multcomp”. The effects of viral infection on foraging onset and recruitment efficiency were analyzed by Pearson chi-square test (*R* package: “nortest”). The significances among the four fragment categories were first analyzed by a one-way ANOVA or Kruskal-Wallis test, and then post hoc by pairwise *T* test or pairwise Kruskal-Wallis test with the Bonferroni correction. All the values were shown by mean ± SD.

For changes in food preference, the full mixed model was built using negative-binomial errors with the total ant numbers at each time point considered as response variables, and colony identity and each time point as random factors. Food types were set as dummy factors, and the interaction effects of food types and infection status or the presence of queen were analyzed using the function “glmer” implemented in “lme4” package (*R* Development Core Team, 2014). The likelihood ratio test was used to determine significance level of each fixed effect by reducing the full model. The relationship between food preference and fragment categories was analyzed using a local regression (locally weighted scatterplot smoothing curve, LOWESS curve). Food preference was ranked, and the significances for each pairwise comparison of the four food resources were determined using the Tukey’s honesty significant difference (HSD) test.

## Electronic supplementary material

Supplementary Dataset 1
