# Supplementary material for: Viral infections in fire ants lead to reduced foraging activity and dietary changes
Source: Sci Rep. 2018 Sep 10;8:13498. doi: 10.1038/s41598-018-31969-3 (PMC6131164; doi:10.1038/s41598-018-31969-3)
Supplement: Supplementary file 1 — Supplementary Dataset 1 [file 41598_2018_31969_MOESM1_ESM.docx]

Supplementary Information

**Viral infections in fire ants lead to reduced foraging activity and dietary changes**

Hung-Wei Hsu^1,2^ ∙ Ming-Chung Chiu^2,3^ ∙ DeWayne Shoemaker^4^ ∙ Chin-Cheng Scotty Yang^5*^

^1^ Graduate School of Agriculture, Kyoto University, Kyoto 606-8502, Japan

^2^ Department of Entomology, National Taiwan University, Taipei 106, Taiwan

^3^ Department of Biological Resources, National Chiayi University, Chiayi 600, Taiwan

^4^ Department of Entomology & Plant Pathology, University of Tennessee, Knoxville, Tennessee, 37996, USA

^5^ Research Institute for Sustainable Humanosphere, Kyoto University, Gokasho, Uji, Kyoto, 611-0011, Japan

^*^ Corresponding author: Chin-Cheng Scotty Yang ([ccyang@rish.kyoto-u.ac.jp](mailto:ccyang@rish.kyoto-u.ac.jp))

**Appendix S1**

Table S1 Colony fragment profiles (geographic information, queen and infection status)

| Colony code | Collection site | Queen located | |
| --- | --- | --- | --- |
|  |  | uninfected | Infected |
| Si-308 | New Taipei City |  | √ |
| Si-309 | New Taipei City | √ |  |
| Si-310 | New Taipei City |  | √ |
| Si-304 | Taoyuan City |  | √ |
| Si-336 | Taoyuan City |  | √ |
| Si-339 | Taoyuan City | √ |  |
| Si-331 | New Taipei City | √ |  |
| Si-342 | Taoyuan city |  | √ |
| Si-326 | New Taipei City | √ |  |

Table S2 Macronutrient composition of the four food resource for food preference test (%)

|  | Carbohydrate | Protein | Lipid | Others (minor) |
| --- | --- | --- | --- | --- |
| Honey | 82.4 | 0.3 | 0 | 17.3 |
| Potato chip | 59.7 | 4.8 | 30.5 | 5 |
| Tuna | 14.5 | 36.2 | 40 | 9.3 |
| Peanut butter | 20 | 25 | 50 | 5 |

Table S3 The pairwise comparisons of foraging intensity, time required to foraging onset and recruitment efficiency

|  | **Uninfected**  **w/ queen** | **Uninfected**  **w/o queen** | **Infected**  **w/ queen** | **Infected**  **w/o queen** |
| --- | --- | --- | --- | --- |
| Foraging intensity (n) | 14778.75±4894.11^a^ | 14004.80±10813.80^a^ | 921.40±446.12^b^ | 613.25±417.10^b^ |
| Onset of foraging (min)^1^ | 17.50±9.57 | 18.00±13.04 | 310.00±504.58 | 135.00±113.58 |
| Recruitment efficiency (min) | 85.00±36.97^a^ | 92.00±44.94^a^ | 622.00±501.47^b^ | 1452.50±169.39^c^ |

^1^ The pairwise comparison was not preformed among the time required to foraging onset since the differences is not significant in Kruskal-Wallis test.

^a–d^ Letters indicate significant pairwise differences (multiple comparisons conducted using a Tukey's honestly significant difference test (foraging intensity) and pairwise *T* test (recruitment efficiency), *P* < 0.05) across the various categories for each segment.
